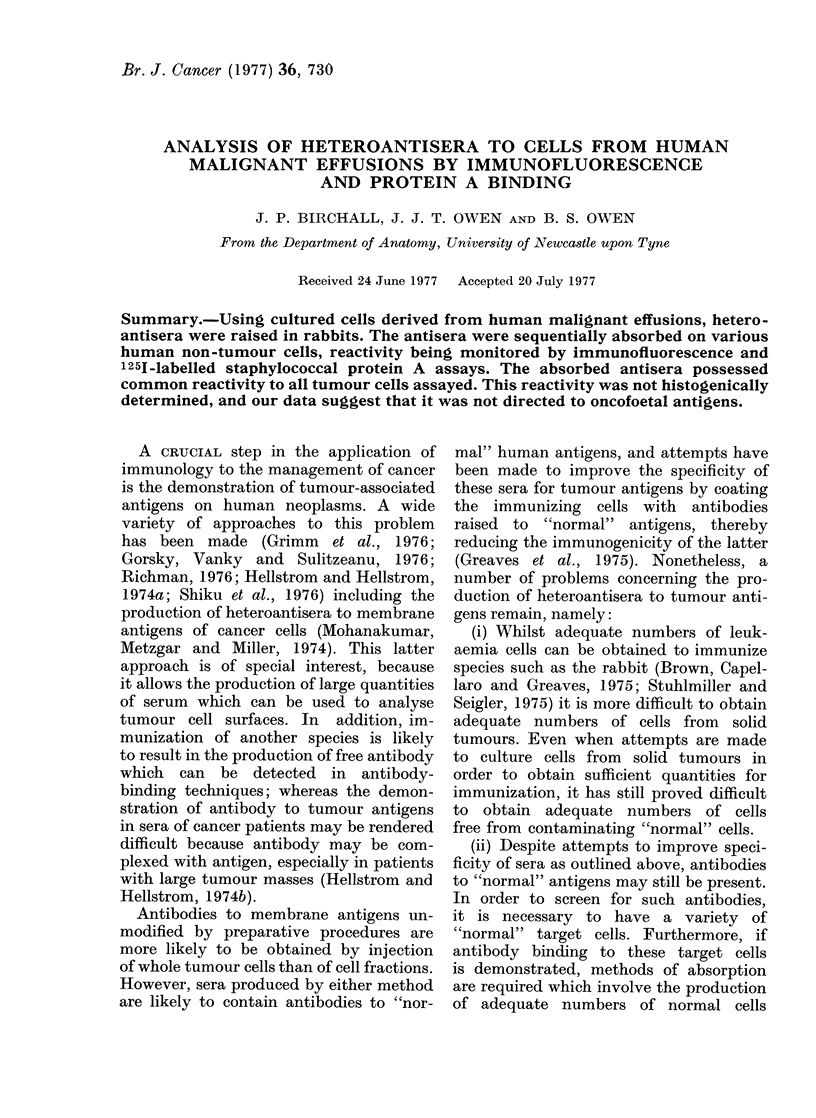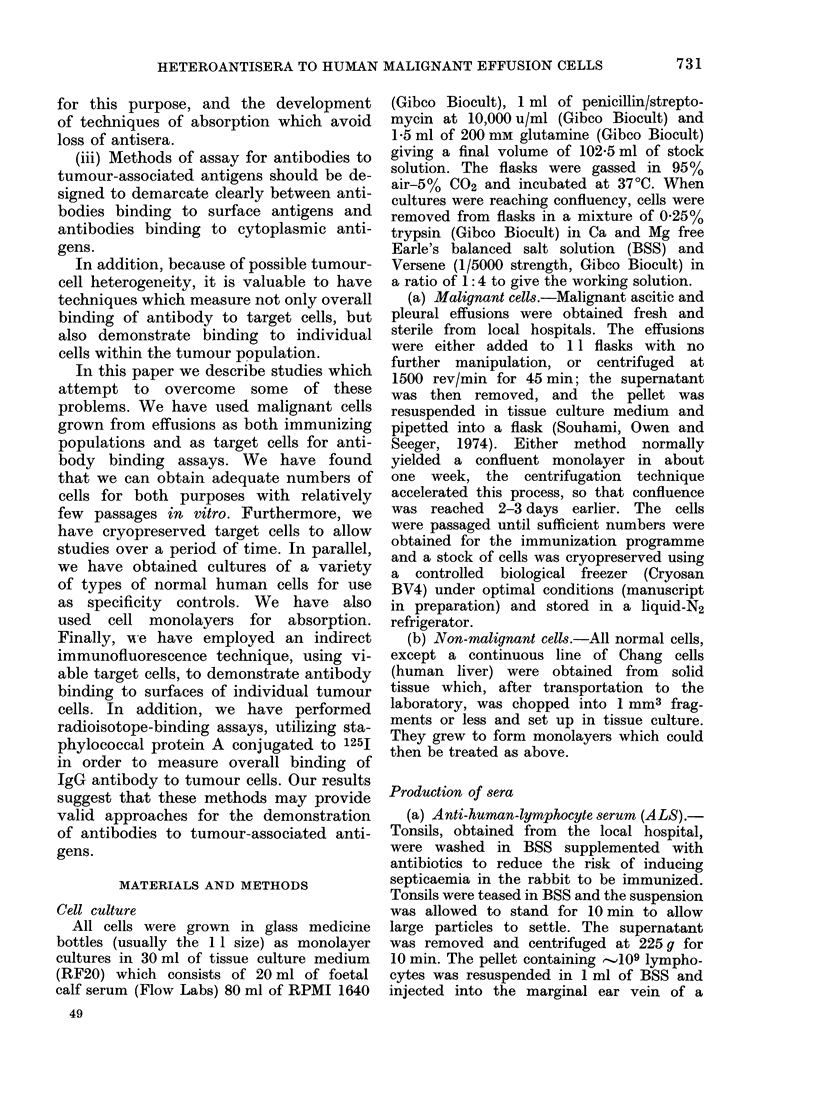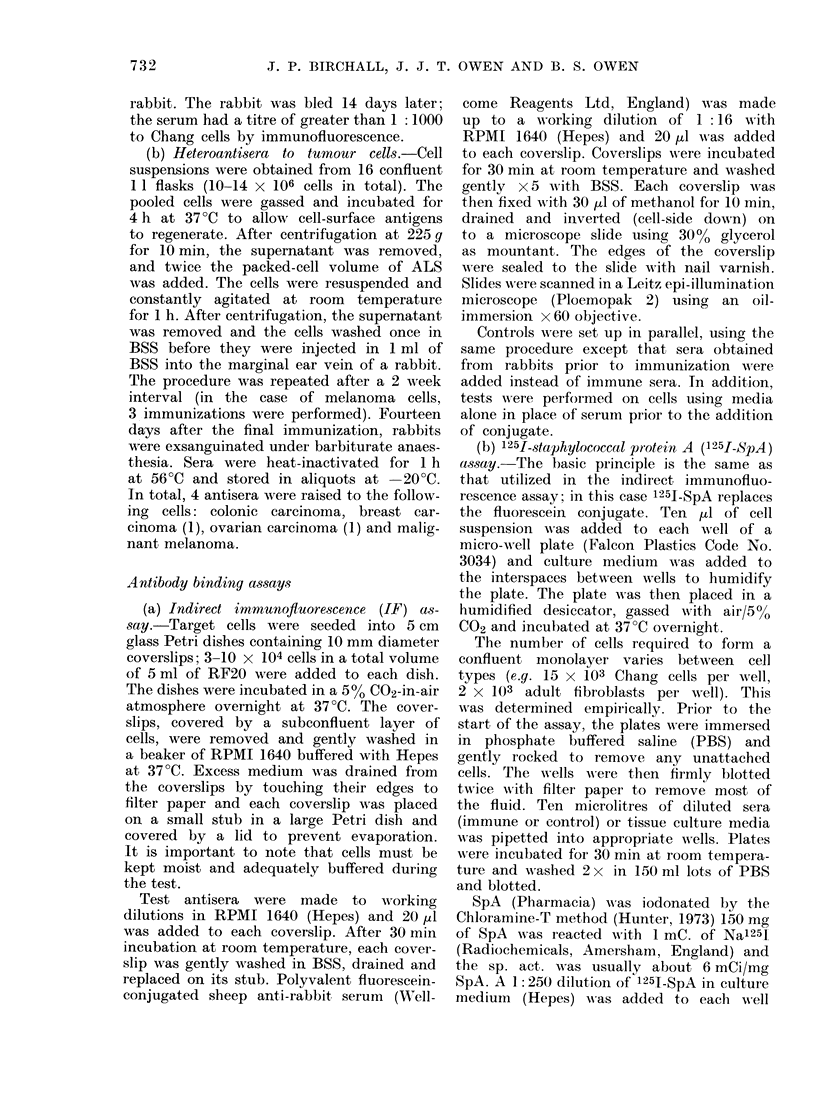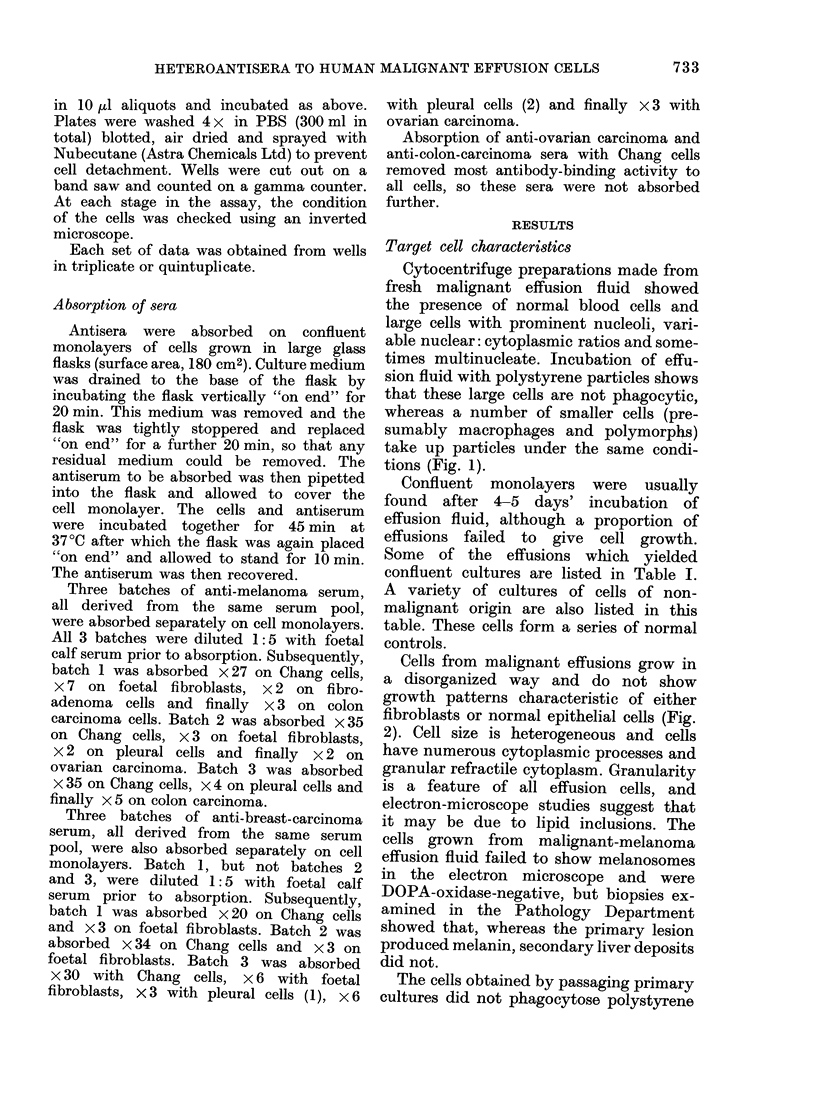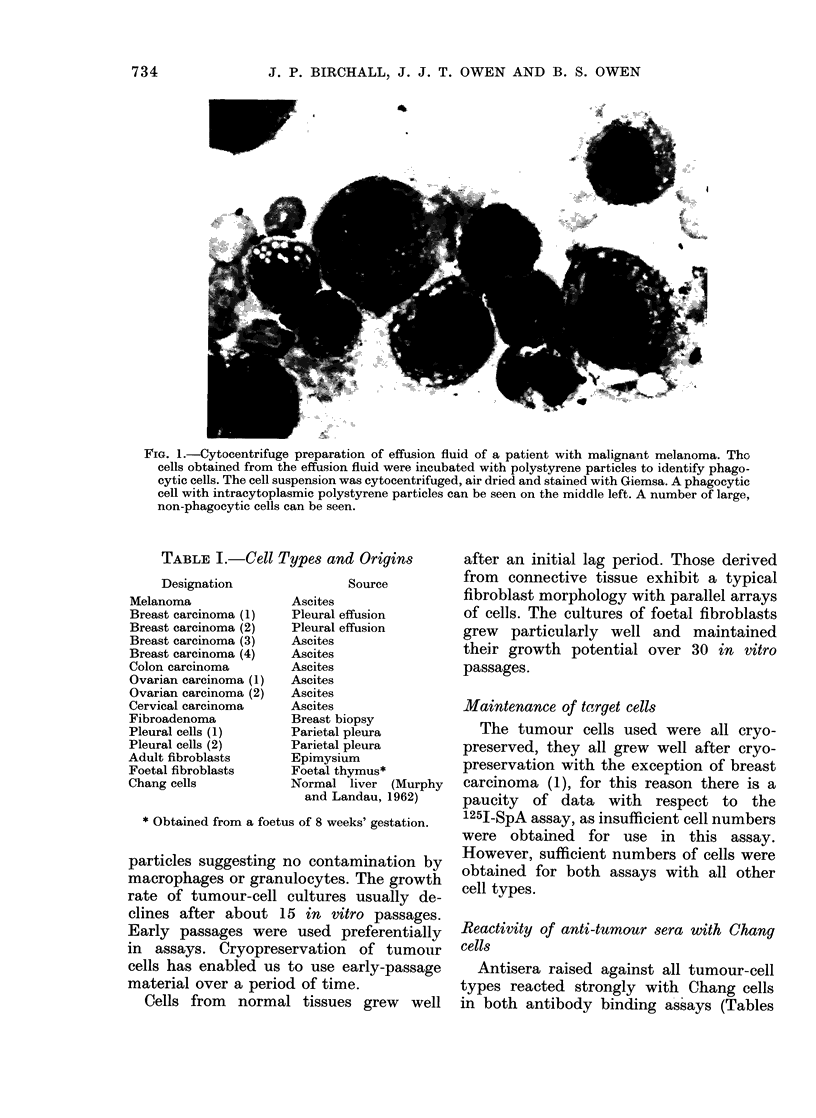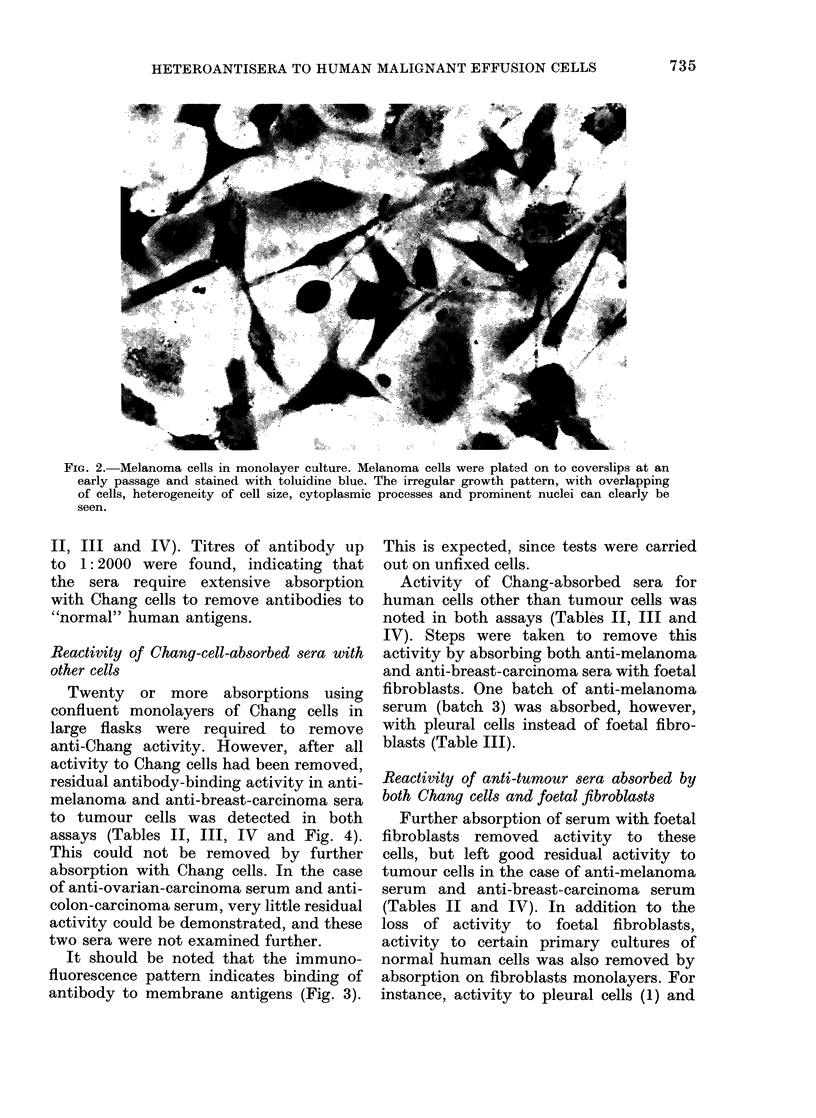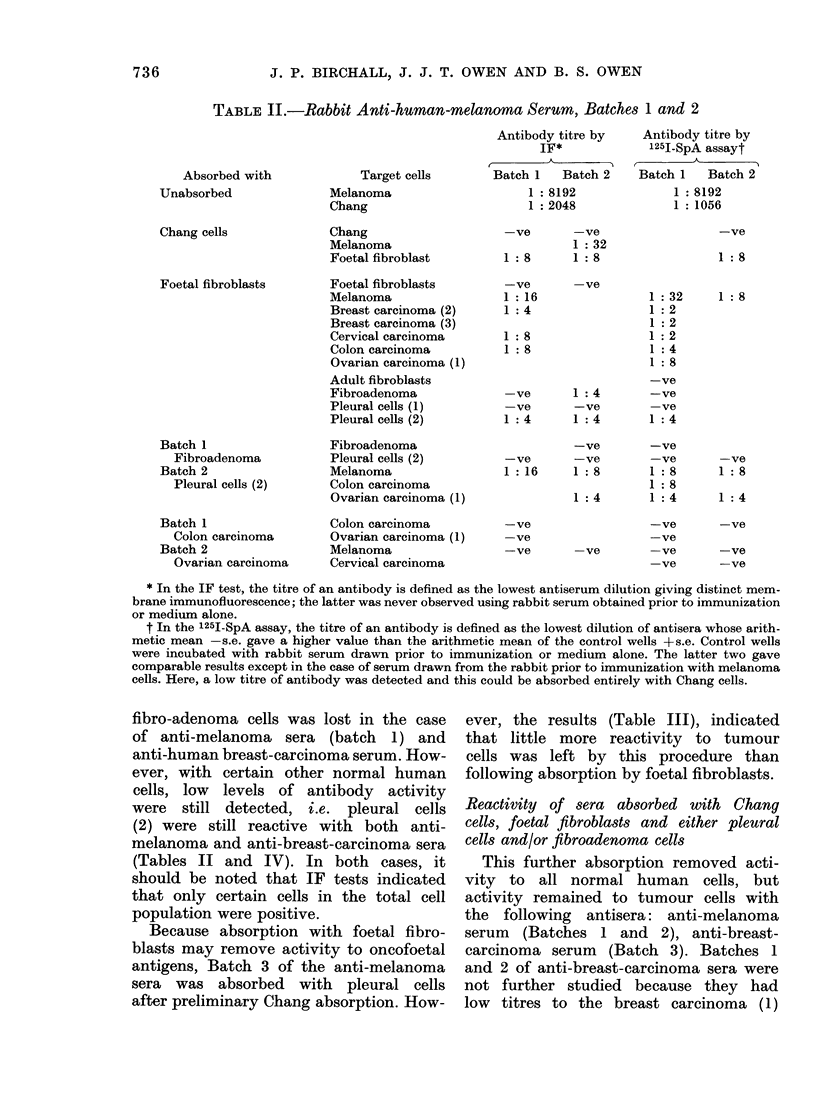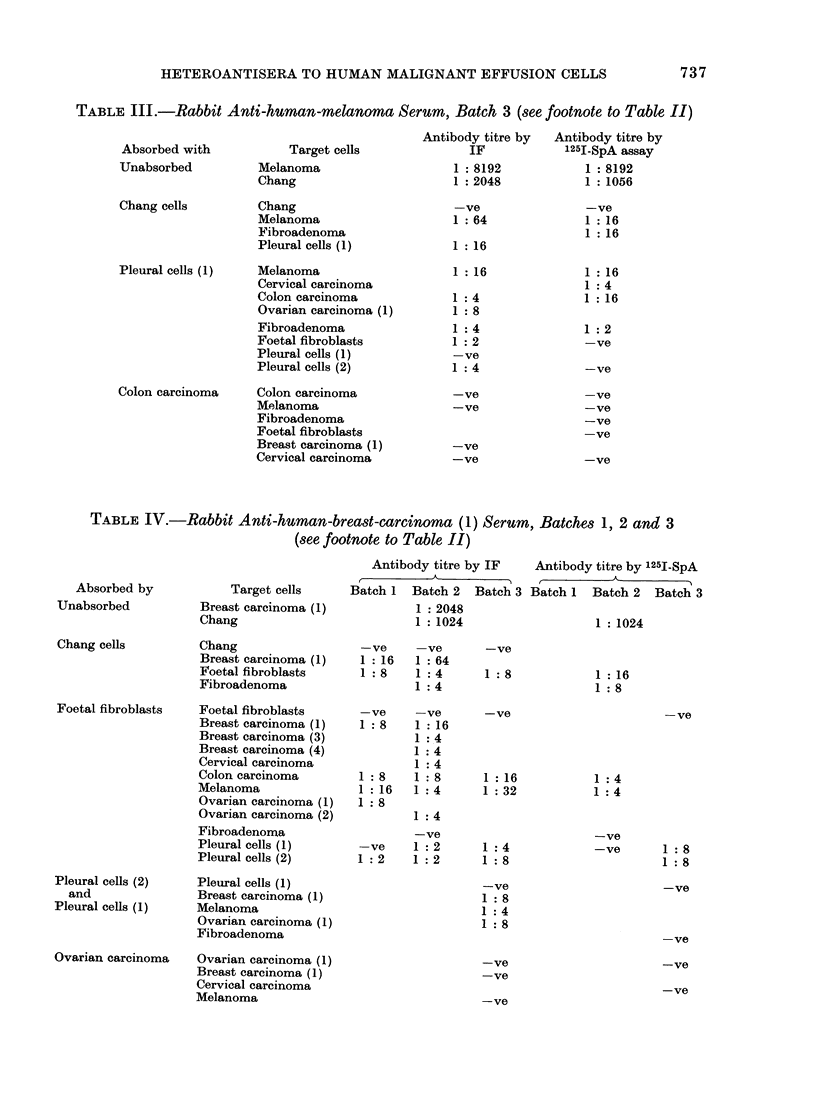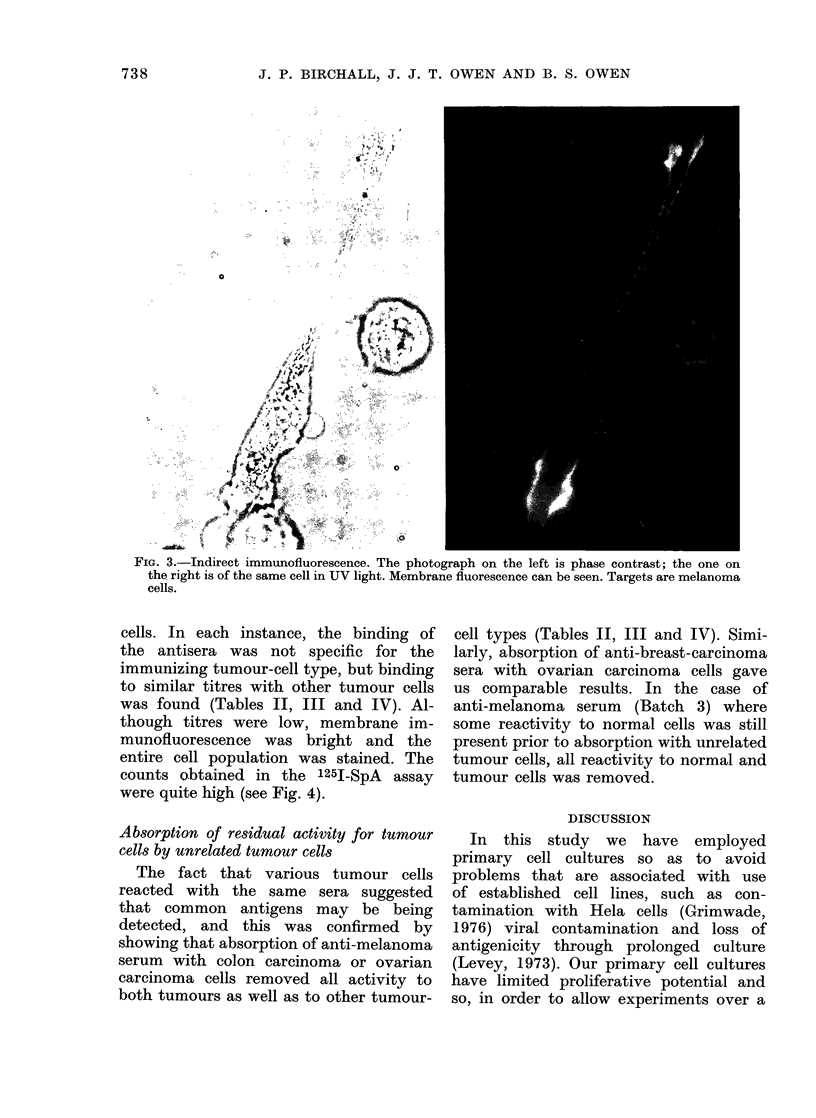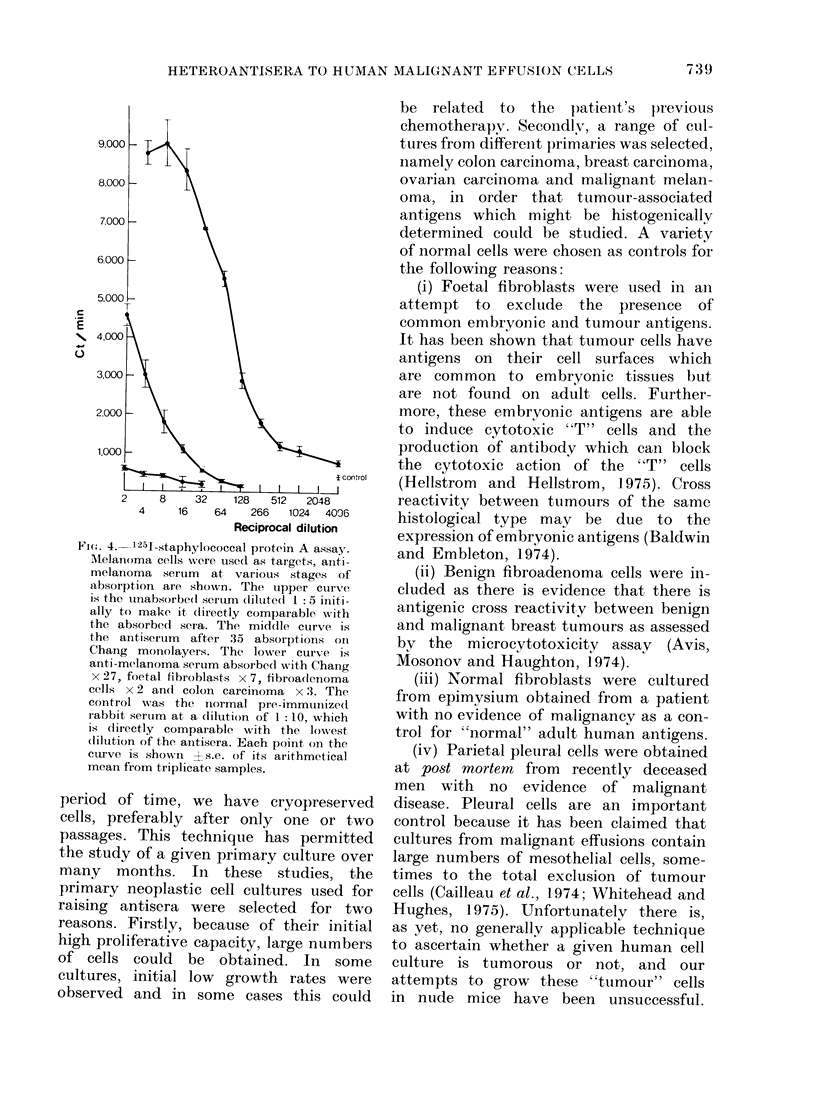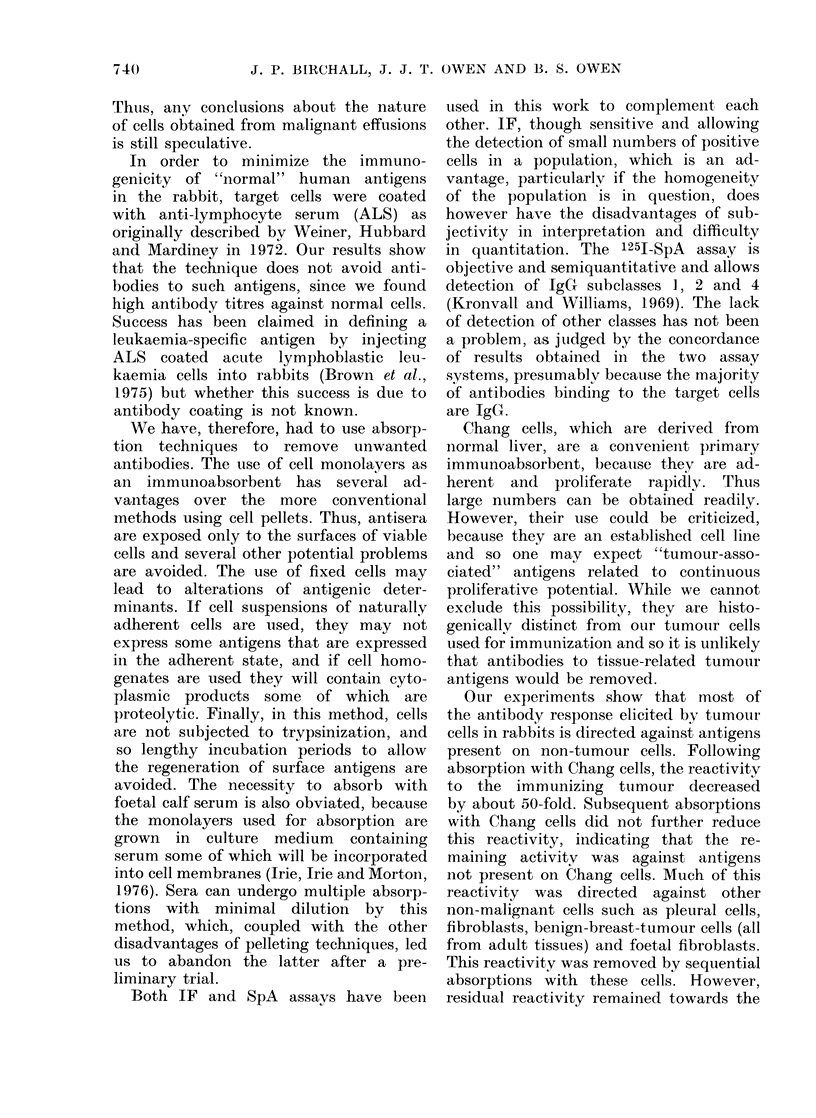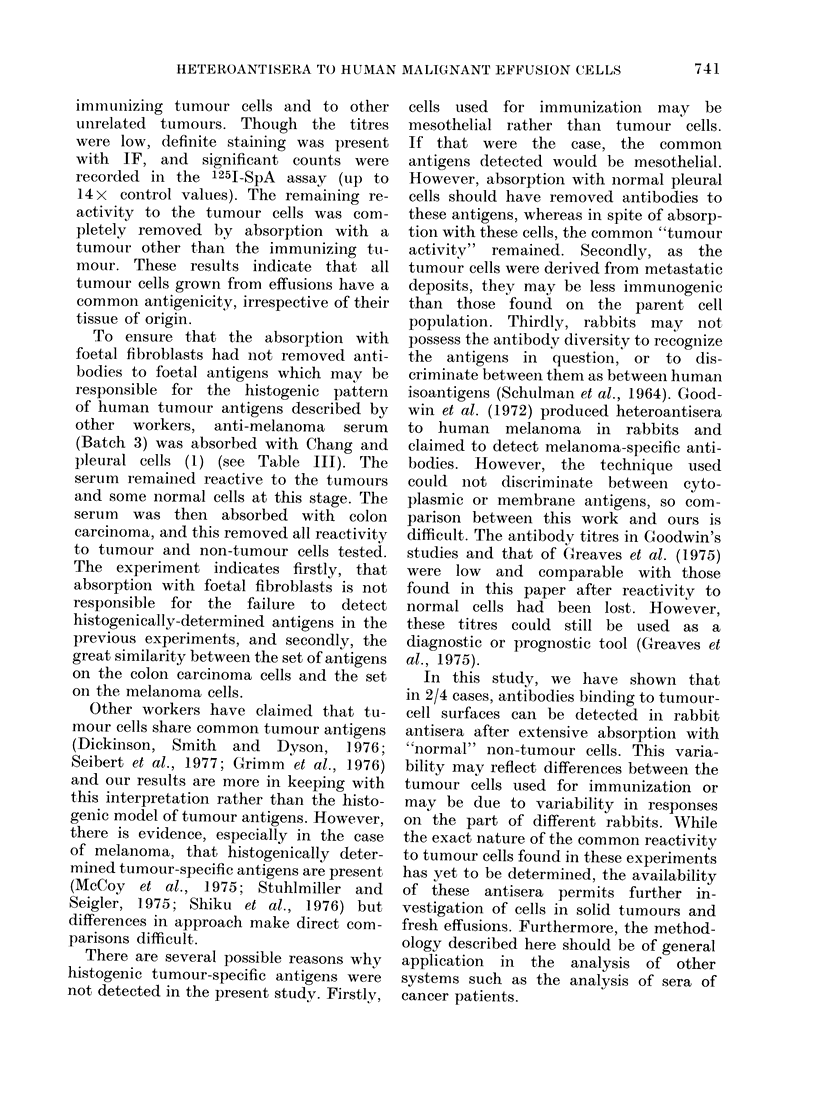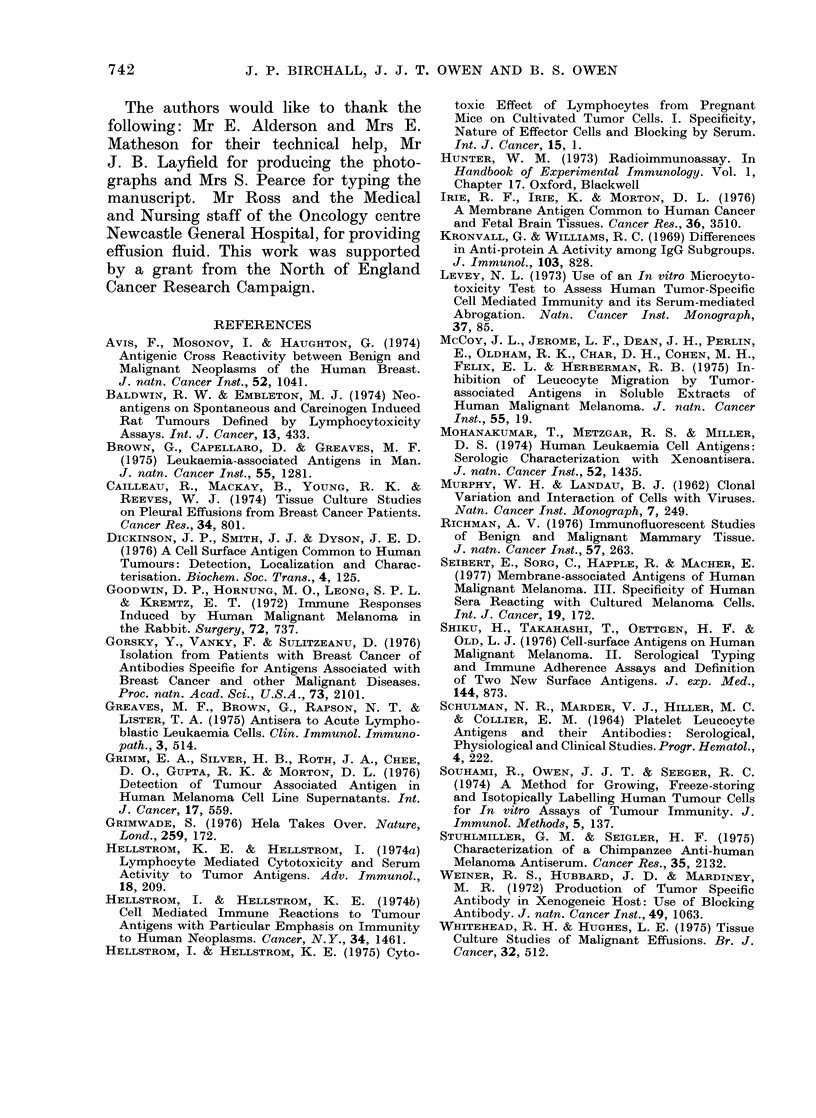# Analysis of heteroantisera to cells from human malignant effusions by immunofluorescence and protein A binding.

**DOI:** 10.1038/bjc.1977.256

**Published:** 1977-12

**Authors:** J. P. Birchall, J. J. Owen, B. S. Owen

## Abstract

**Images:**


*Br. J. Cancer* (1977) **36**, 730

### ANALYSIS OF HETEROANTISERA TO CELLS FROM HUMAN MALIGNANT EFFUSIONS BY IMMUNOFLUORESCENCE AND PROTEIN A BINDING

J. P. BIRCHALL, J. J. T. OWEN AND B. S. OWEN

*From the Department of Anatomy, University of Newcastle upon Tyne*

Received 24 June 1977 Accepted 20 July 1977

**Summary.—Using cultured cells derived from human malignant effusions, heteroantisera were raised in rabbits. The antisera were sequentially absorbed on various human non-tumour cells, reactivity being monitored by immunofluorescence and $^{125}$I-labelled staphylococcal protein A assays. The absorbed antisera possessed common reactivity to all tumour cells assayed. This reactivity was not histogenically determined, and our data suggest that it was not directed to oncofoetal antigens.**

A CRUCIAL step in the application of immunology to the management of cancer is the demonstration of tumour-associated antigens on human neoplasms. A wide variety of approaches to this problem has been made (Grimm *et al.*, 1976; Gorsky, Vanky and Sulitzeanu, 1976; Richman, 1976; Hellstrom and Hellstrom, 1974*a*; Shiku *et al.*, 1976) including the production of heteroantisera to membrane antigens of cancer cells (Mohanakumar, Metzgar and Miller, 1974). This latter approach is of special interest, because it allows the production of large quantities of serum which can be used to analyse tumour cell surfaces. In addition, immunization of another species is likely to result in the production of free antibody which can be detected in antibody-binding techniques; whereas the demonstration of antibody to tumour antigens in sera of cancer patients may be rendered difficult because antibody may be complexed with antigen, especially in patients with large tumour masses (Hellstrom and Hellstrom, 1974*b*).

Antibodies to membrane antigens unmodified by preparative procedures are more likely to be obtained by injection of whole tumour cells than of cell fractions. However, sera produced by either method are likely to contain antibodies to "normal" human antigens, and attempts have been made to improve the specificity of these sera for tumour antigens by coating the immunizing cells with antibodies raised to "normal" antigens, thereby reducing the immunogenicity of the latter (Greaves *et al.*, 1975). Nonetheless, a number of problems concerning the production of heteroantisera to tumour antigens remain, namely:

(i) Whilst adequate numbers of leukaemia cells can be obtained to immunize species such as the rabbit (Brown, Capellaro and Greaves, 1975; Stuhlmiller and Seigler, 1975) it is more difficult to obtain adequate numbers of cells from solid tumours. Even when attempts are made to culture cells from solid tumours in order to obtain sufficient quantities for immunization, it has still proved difficult to obtain adequate numbers of cells free from contaminating "normal" cells.

(ii) Despite attempts to improve specificity of sera as outlined above, antibodies to "normal" antigens may still be present. In order to screen for such antibodies, it is necessary to have a variety of "normal" target cells. Furthermore, if antibody binding to these target cells is demonstrated, methods of absorption are required which involve the production of adequate numbers of normal cells for this purpose, and the development of techniques of absorption which avoid loss of antisera.

(iii) Methods of assay for antibodies to tumour-associated antigens should be designed to demarcate clearly between antibodies binding to surface antigens and antibodies binding to cytoplasmic antigens.

In addition, because of possible tumour-cell heterogeneity, it is valuable to have techniques which measure not only overall binding of antibody to target cells, but also demonstrate binding to individual cells within the tumour population.

In this paper we describe studies which attempt to overcome some of these problems. We have used malignant cells grown from effusions as both immunizing populations and as target cells for antibody binding assays. We have found that we can obtain adequate numbers of cells for both purposes with relatively few passages *in vitro*. Furthermore, we have cryopreserved target cells to allow studies over a period of time. In parallel, we have obtained cultures of a variety of types of normal human cells for use as specificity controls. We have also used cell monolayers for absorption. Finally, we have employed an indirect immunofluorescence technique, using viable target cells, to demonstrate antibody binding to surfaces of individual tumour cells. In addition, we have performed radioisotope-binding assays, utilizing staphylococcal protein A conjugated to $^{125}I$ in order to measure overall binding of IgG antibody to tumour cells. Our results suggest that these methods may provide valid approaches for the demonstration of antibodies to tumour-associated antigens.

### MATERIALS AND METHODS

### *Cell culture*

All cells were grown in glass medicine bottles (usually the 1 l size) as monolayer cultures in 30 ml of tissue culture medium (RF20) which consists of 20 ml of foetal calf serum (Flow Labs) 80 ml of RPMI 1640 (Gibco Biocult), 1 ml of penicillin/streptomycin at 10,000 u/ml (Gibco Biocult) and 1·5 ml of 200 mM glutamine (Gibco Biocult) giving a final volume of 102·5 ml of stock solution. The flasks were gassed in 95% air–5% $CO_2$ and incubated at 37°C. When cultures were reaching confluency, cells were removed from flasks in a mixture of 0·25% trypsin (Gibco Biocult) in Ca and Mg free Earle's balanced salt solution (BSS) and Versene (1/5000 strength, Gibco Biocult) in a ratio of 1 : 4 to give the working solution.

(a) *Malignant cells.*—Malignant ascitic and pleural effusions were obtained fresh and sterile from local hospitals. The effusions were either added to 1 l flasks with no further manipulation, or centrifuged at 1500 rev/min for 45 min; the supernatant was then removed, and the pellet was resuspended in tissue culture medium and pipetted into a flask (Souhami, Owen and Seeger, 1974). Either method normally yielded a confluent monolayer in about one week, the centrifugation technique accelerated this process, so that confluence was reached 2–3 days earlier. The cells were passaged until sufficient numbers were obtained for the immunization programme and a stock of cells was cryopreserved using a controlled biological freezer (Cryosan BV4) under optimal conditions (manuscript in preparation) and stored in a liquid-$N_2$ refrigerator.

(b) *Non-malignant cells.*—All normal cells, except a continuous line of Chang cells (human liver) were obtained from solid tissue which, after transportation to the laboratory, was chopped into 1 $mm^3$ fragments or less and set up in tissue culture. They grew to form monolayers which could then be treated as above.

### *Production of sera*

(a) *Anti-human-lymphocyte serum* (*ALS*).—Tonsils, obtained from the local hospital, were washed in BSS supplemented with antibiotics to reduce the risk of inducing septicaemia in the rabbit to be immunized. Tonsils were teased in BSS and the suspension was allowed to stand for 10 min to allow large particles to settle. The supernatant was removed and centrifuged at 225 *g* for 10 min. The pellet containing $\sim 10^9$ lymphocytes was resuspended in 1 ml of BSS and injected into the marginal ear vein of a rabbit. The rabbit was bled 14 days later; the serum had a titre of greater than 1 : 1000 to Chang cells by immunofluorescence.

(b) *Heteroantisera to tumour cells.*—Cell suspensions were obtained from 16 confluent 1 l flasks (10–14 × $10^6$ cells in total). The pooled cells were gassed and incubated for 4 h at 37°C to allow cell-surface antigens to regenerate. After centrifugation at 225 *g* for 10 min, the supernatant was removed, and twice the packed-cell volume of ALS was added. The cells were resuspended and constantly agitated at room temperature for 1 h. After centrifugation, the supernatant was removed and the cells washed once in BSS before they were injected in 1 ml of BSS into the marginal ear vein of a rabbit. The procedure was repeated after a 2 week interval (in the case of melanoma cells, 3 immunizations were performed). Fourteen days after the final immunization, rabbits were exsanguinated under barbiturate anaesthesia. Sera were heat-inactivated for 1 h at 56°C and stored in aliquots at −20°C. In total, 4 antisera were raised to the following cells: colonic carcinoma, breast carcinoma (1), ovarian carcinoma (1) and malignant melanoma.

*Antibody binding assays*

(a) *Indirect immunofluorescence* (*IF*) *assay.*—Target cells were seeded into 5 cm glass Petri dishes containing 10 mm diameter coverslips; 3–10 × $10^4$ cells in a total volume of 5 ml of RF20 were added to each dish. The dishes were incubated in a 5% $CO_2$-in-air atmosphere overnight at 37°C. The coverslips, covered by a subconfluent layer of cells, were removed and gently washed in a beaker of RPMI 1640 buffered with Hepes at 37°C. Excess medium was drained from the coverslips by touching their edges to filter paper and each coverslip was placed on a small stub in a large Petri dish and covered by a lid to prevent evaporation. It is important to note that cells must be kept moist and adequately buffered during the test.

Test antisera were made to working dilutions in RPMI 1640 (Hepes) and 20 μl was added to each coverslip. After 30 min incubation at room temperature, each coverslip was gently washed in BSS, drained and replaced on its stub. Polyvalent fluorescein-conjugated sheep anti-rabbit serum (Wellcome Reagents Ltd, England) was made up to a working dilution of 1 : 16 with RPMI 1640 (Hepes) and 20 μl was added to each coverslip. Coverslips were incubated for 30 min at room temperature and washed gently ×5 with BSS. Each coverslip was then fixed with 30 μl of methanol for 10 min, drained and inverted (cell-side down) on to a microscope slide using 30% glycerol as mountant. The edges of the coverslip were sealed to the slide with nail varnish. Slides were scanned in a Leitz epi-illumination microscope (Ploemopak 2) using an oil-immersion ×60 objective.

Controls were set up in parallel, using the same procedure except that sera obtained from rabbits prior to immunization were added instead of immune sera. In addition, tests were performed on cells using media alone in place of serum prior to the addition of conjugate.

(b) *$^{125}$I-staphylococcal protein A* (*$^{125}$I-SpA*) *assay.*—The basic principle is the same as that utilized in the indirect immunofluorescence assay; in this case $^{125}$I-SpA replaces the fluorescein conjugate. Ten μl of cell suspension was added to each well of a micro-well plate (Falcon Plastics Code No. 3034) and culture medium was added to the interspaces between wells to humidify the plate. The plate was then placed in a humidified desiccator, gassed with air/5% $CO_2$ and incubated at 37°C overnight.

The number of cells required to form a confluent monolayer varies between cell types (*e.g.* 15 × $10^3$ Chang cells per well, 2 × $10^3$ adult fibroblasts per well). This was determined empirically. Prior to the start of the assay, the plates were immersed in phosphate buffered saline (PBS) and gently rocked to remove any unattached cells. The wells were then firmly blotted twice with filter paper to remove most of the fluid. Ten microlitres of diluted sera (immune or control) or tissue culture media was pipetted into appropriate wells. Plates were incubated for 30 min at room temperature and washed 2× in 150 ml lots of PBS and blotted.

SpA (Pharmacia) was iodonated by the Chloramine-T method (Hunter, 1973) 150 mg of SpA was reacted with 1 mC. of $Na^{125}I$ (Radiochemicals, Amersham, England) and the sp. act. was usually about 6 mCi/mg SpA. A 1 : 250 dilution of $^{125}$I-SpA in culture medium (Hepes) was added to each well in 10 $\mu$l aliquots and incubated as above. Plates were washed 4× in PBS (300 ml in total) blotted, air dried and sprayed with Nubecutane (Astra Chemicals Ltd) to prevent cell detachment. Wells were cut out on a band saw and counted on a gamma counter. At each stage in the assay, the condition of the cells was checked using an inverted microscope.

Each set of data was obtained from wells in triplicate or quintuplicate.

*Absorption of sera*

Antisera were absorbed on confluent monolayers of cells grown in large glass flasks (surface area, 180 cm$^2$). Culture medium was drained to the base of the flask by incubating the flask vertically "on end" for 20 min. This medium was removed and the flask was tightly stoppered and replaced "on end" for a further 20 min, so that any residual medium could be removed. The antiserum to be absorbed was then pipetted into the flask and allowed to cover the cell monolayer. The cells and antiserum were incubated together for 45 min at 37°C after which the flask was again placed "on end" and allowed to stand for 10 min. The antiserum was then recovered.

Three batches of anti-melanoma serum, all derived from the same serum pool, were absorbed separately on cell monolayers. All 3 batches were diluted 1:5 with foetal calf serum prior to absorption. Subsequently, batch 1 was absorbed ×27 on Chang cells, ×7 on foetal fibroblasts, ×2 on fibro-adenoma cells and finally ×3 on colon carcinoma cells. Batch 2 was absorbed ×35 on Chang cells, ×3 on foetal fibroblasts, ×2 on pleural cells and finally ×2 on ovarian carcinoma. Batch 3 was absorbed ×35 on Chang cells, ×4 on pleural cells and finally ×5 on colon carcinoma.

Three batches of anti-breast-carcinoma serum, all derived from the same serum pool, were also absorbed separately on cell monolayers. Batch 1, but not batches 2 and 3, were diluted 1:5 with foetal calf serum prior to absorption. Subsequently, batch 1 was absorbed ×20 on Chang cells and ×3 on foetal fibroblasts. Batch 2 was absorbed ×34 on Chang cells and ×3 on foetal fibroblasts. Batch 3 was absorbed ×30 with Chang cells, ×6 with foetal fibroblasts, ×3 with pleural cells (1), ×6 with pleural cells (2) and finally ×3 with ovarian carcinoma.

Absorption of anti-ovarian carcinoma and anti-colon-carcinoma sera with Chang cells removed most antibody-binding activity to all cells, so these sera were not absorbed further.

### RESULTS

*Target cell characteristics*

Cytocentrifuge preparations made from fresh malignant effusion fluid showed the presence of normal blood cells and large cells with prominent nucleoli, variable nuclear: cytoplasmic ratios and sometimes multinucleate. Incubation of effusion fluid with polystyrene particles shows that these large cells are not phagocytic, whereas a number of smaller cells (presumably macrophages and polymorphs) take up particles under the same conditions (Fig. 1).

Confluent monolayers were usually found after 4–5 days' incubation of effusion fluid, although a proportion of effusions failed to give cell growth. Some of the effusions which yielded confluent cultures are listed in Table I. A variety of cultures of cells of non-malignant origin are also listed in this table. These cells form a series of normal controls.

Cells from malignant effusions grow in a disorganized way and do not show growth patterns characteristic of either fibroblasts or normal epithelial cells (Fig. 2). Cell size is heterogeneous and cells have numerous cytoplasmic processes and granular refractile cytoplasm. Granularity is a feature of all effusion cells, and electron-microscope studies suggest that it may be due to lipid inclusions. The cells grown from malignant-melanoma effusion fluid failed to show melanosomes in the electron microscope and were DOPA-oxidase-negative, but biopsies examined in the Pathology Department showed that, whereas the primary lesion produced melanin, secondary liver deposits did not.

The cells obtained by passaging primary cultures did not phagocytose polystyrene

FIG. 1.—Cytocentrifuge preparation of effusion fluid of a patient with malignant melanoma. The cells obtained from the effusion fluid were incubated with polystyrene particles to identify phagocytic cells. The cell suspension was cytocentrifuged, air dried and stained with Giemsa. A phagocytic cell with intracytoplasmic polystyrene particles can be seen on the middle left. A number of large, non-phagocytic cells can be seen.

TABLE I.—*Cell Types and Origins*

| Designation | Source |
|---|---|
| Melanoma | Ascites |
| Breast carcinoma (1) | Pleural effusion |
| Breast carcinoma (2) | Pleural effusion |
| Breast carcinoma (3) | Ascites |
| Breast carcinoma (4) | Ascites |
| Colon carcinoma | Ascites |
| Ovarian carcinoma (1) | Ascites |
| Ovarian carcinoma (2) | Ascites |
| Cervical carcinoma | Ascites |
| Fibroadenoma | Breast biopsy |
| Pleural cells (1) | Parietal pleura |
| Pleural cells (2) | Parietal pleura |
| Adult fibroblasts | Epimysium |
| Foetal fibroblasts | Foetal thymus* |
| Chang cells | Normal liver (Murphy and Landau, 1962) |

* Obtained from a foetus of 8 weeks' gestation.

particles suggesting no contamination by macrophages or granulocytes. The growth rate of tumour-cell cultures usually declines after about 15 *in vitro* passages. Early passages were used preferentially in assays. Cryopreservation of tumour cells has enabled us to use early-passage material over a period of time.

Cells from normal tissues grew well after an initial lag period. Those derived from connective tissue exhibit a typical fibroblast morphology with parallel arrays of cells. The cultures of foetal fibroblasts grew particularly well and maintained their growth potential over 30 *in vitro* passages.

*Maintenance of target cells*

The tumour cells used were all cryopreserved, they all grew well after cryopreservation with the exception of breast carcinoma (1), for this reason there is a paucity of data with respect to the $^{125}$I-SpA assay, as insufficient cell numbers were obtained for use in this assay. However, sufficient numbers of cells were obtained for both assays with all other cell types.

*Reactivity of anti-tumour sera with Chang cells*

Antisera raised against all tumour-cell types reacted strongly with Chang cells in both antibody binding assays (Tables

FIG. 2.—Melanoma cells in monolayer culture. Melanoma cells were plated on to coverslips at an early passage and stained with toluidine blue. The irregular growth pattern, with overlapping of cells, heterogeneity of cell size, cytoplasmic processes and prominent nuclei can clearly be seen.

II, III and IV). Titres of antibody up to 1:2000 were found, indicating that the sera require extensive absorption with Chang cells to remove antibodies to "normal" human antigens.

*Reactivity of Chang-cell-absorbed sera with other cells*

Twenty or more absorptions using confluent monolayers of Chang cells in large flasks were required to remove anti-Chang activity. However, after all activity to Chang cells had been removed, residual antibody-binding activity in anti-melanoma and anti-breast-carcinoma sera to tumour cells was detected in both assays (Tables II, III, IV and Fig. 4). This could not be removed by further absorption with Chang cells. In the case of anti-ovarian-carcinoma serum and anti-colon-carcinoma serum, very little residual activity could be demonstrated, and these two sera were not examined further.

It should be noted that the immunofluorescence pattern indicates binding of antibody to membrane antigens (Fig. 3). This is expected, since tests were carried out on unfixed cells.

Activity of Chang-absorbed sera for human cells other than tumour cells was noted in both assays (Tables II, III and IV). Steps were taken to remove this activity by absorbing both anti-melanoma and anti-breast-carcinoma sera with foetal fibroblasts. One batch of anti-melanoma serum (batch 3) was absorbed, however, with pleural cells instead of foetal fibroblasts (Table III).

*Reactivity of anti-tumour sera absorbed by both Chang cells and foetal fibroblasts*

Further absorption of serum with foetal fibroblasts removed activity to these cells, but left good residual activity to tumour cells in the case of anti-melanoma serum and anti-breast-carcinoma serum (Tables II and IV). In addition to the loss of activity to foetal fibroblasts, activity to certain primary cultures of normal human cells was also removed by absorption on fibroblasts monolayers. For instance, activity to pleural cells (1) and

TABLE II.—*Rabbit Anti-human-melanoma Serum, Batches* 1 *and* 2

| Absorbed with | Target cells | Antibody titre by IF* | | Antibody titre by $^{125}$I-SpA assay† | |
|---|---|---|---|---|---|
| | | Batch 1 | Batch 2 | Batch 1 | Batch 2 |
| Unabsorbed | Melanoma | 1 : 8192 | | 1 : 8192 | |
| | Chang | 1 : 2048 | | 1 : 1056 | |
| Chang cells | Chang | —ve | —ve | | —ve |
| | Melanoma | | 1 : 32 | | |
| | Foetal fibroblast | 1 : 8 | 1 : 8 | | 1 : 8 |
| Foetal fibroblasts | Foetal fibroblasts | —ve | —ve | | |
| | Melanoma | 1 : 16 | | 1 : 32 | 1 : 8 |
| | Breast carcinoma (2) | 1 : 4 | | 1 : 2 | |
| | Breast carcinoma (3) | | | 1 : 2 | |
| | Cervical carcinoma | 1 : 8 | | 1 : 2 | |
| | Colon carcinoma | 1 : 8 | | 1 : 4 | |
| | Ovarian carcinoma (1) | | | 1 : 8 | |
| | Adult fibroblasts | | | —ve | |
| | Fibroadenoma | —ve | 1 : 4 | —ve | |
| | Pleural cells (1) | —ve | —ve | —ve | |
| | Pleural cells (2) | 1 : 4 | 1 : 4 | 1 : 4 | |
| Batch 1 | Fibroadenoma | | —ve | —ve | |
| Fibroadenoma | Pleural cells (2) | —ve | —ve | —ve | —ve |
| Batch 2 | Melanoma | 1 : 16 | 1 : 8 | 1 : 8 | 1 : 8 |
| Pleural cells (2) | Colon carcinoma | | | 1 : 8 | |
| | Ovarian carcinoma (1) | | 1 : 4 | 1 : 4 | 1 : 4 |
| Batch 1 | Colon carcinoma | —ve | | —ve | —ve |
| Colon carcinoma | Ovarian carcinoma (1) | —ve | | —ve | |
| Batch 2 | Melanoma | —ve | —ve | —ve | —ve |
| Ovarian carcinoma | Cervical carcinoma | | | —ve | —ve |

* In the IF test, the titre of an antibody is defined as the lowest antiserum dilution giving distinct membrane immunofluorescence; the latter was never observed using rabbit serum obtained prior to immunization or medium alone.

† In the $^{125}$I-SpA assay, the titre of an antibody is defined as the lowest dilution of antisera whose arithmetic mean —s.e. gave a higher value than the arithmetic mean of the control wells +s.e. Control wells were incubated with rabbit serum drawn prior to immunization or medium alone. The latter two gave comparable results except in the case of serum drawn from the rabbit prior to immunization with melanoma cells. Here, a low titre of antibody was detected and this could be absorbed entirely with Chang cells.

fibro-adenoma cells was lost in the case of anti-melanoma sera (batch 1) and anti-human breast-carcinoma serum. However, with certain other normal human cells, low levels of antibody activity were still detected, *i.e.* pleural cells (2) were still reactive with both anti-melanoma and anti-breast-carcinoma sera (Tables II and IV). In both cases, it should be noted that IF tests indicated that only certain cells in the total cell population were positive.

Because absorption with foetal fibroblasts may remove activity to oncofoetal antigens, Batch 3 of the anti-melanoma sera was absorbed with pleural cells after preliminary Chang absorption. However, the results (Table III), indicated that little more reactivity to tumour cells was left by this procedure than following absorption by foetal fibroblasts.

*Reactivity of sera absorbed with Chang cells, foetal fibroblasts and either pleural cells and/or fibroadenoma cells*

This further absorption removed activity to all normal human cells, but activity remained to tumour cells with the following antisera: anti-melanoma serum (Batches 1 and 2), anti-breast-carcinoma serum (Batch 3). Batches 1 and 2 of anti-breast-carcinoma sera were not further studied because they had low titres to the breast carcinoma (1)

TABLE III.—*Rabbit Anti-human-melanoma Serum, Batch* 3 (*see footnote to Table II*)

| Absorbed with | Target cells | Antibody titre by IF | Antibody titre by $^{125}$I-SpA assay |
|---|---|---|---|
| Unabsorbed | Melanoma | 1 : 8192 | 1 : 8192 |
| | Chang | 1 : 2048 | 1 : 1056 |
| Chang cells | Chang | —ve | —ve |
| | Melanoma | 1 : 64 | 1 : 16 |
| | Fibroadenoma | | 1 : 16 |
| | Pleural cells (1) | 1 : 16 | |
| Pleural cells (1) | Melanoma | 1 : 16 | 1 : 16 |
| | Cervical carcinoma | | 1 : 4 |
| | Colon carcinoma | 1 : 4 | 1 : 16 |
| | Ovarian carcinoma (1) | 1 : 8 | |
| | Fibroadenoma | 1 : 4 | 1 : 2 |
| | Foetal fibroblasts | 1 : 2 | —ve |
| | Pleural cells (1) | —ve | |
| | Pleural cells (2) | 1 : 4 | —ve |
| Colon carcinoma | Colon carcinoma | —ve | —ve |
| | Melanoma | —ve | —ve |
| | Fibroadenoma | | —ve |
| | Foetal fibroblasts | | —ve |
| | Breast carcinoma (1) | —ve | |
| | Cervical carcinoma | —ve | —ve |

TABLE IV.—*Rabbit Anti-human-breast-carcinoma* (1) *Serum, Batches* 1, 2 *and* 3 (*see footnote to Table II*)

| | | Antibody titre by IF | | | Antibody titre by $^{125}$I-SpA | | |
|---|---|---|---|---|---|---|---|
| Absorbed by | Target cells | Batch 1 | Batch 2 | Batch 3 | Batch 1 | Batch 2 | Batch 3 |
| Unabsorbed | Breast carcinoma (1) | | 1 : 2048 | | | | |
| | Chang | | 1 : 1024 | | | 1 : 1024 | |
| Chang cells | Chang | —ve | —ve | —ve | | | |
| | Breast carcinoma (1) | 1 : 16 | 1 : 64 | | | | |
| | Foetal fibroblasts | 1 : 8 | 1 : 4 | 1 : 8 | | 1 : 16 | |
| | Fibroadenoma | | 1 : 4 | | | 1 : 8 | |
| Foetal fibroblasts | Foetal fibroblasts | —ve | —ve | —ve | | | —ve |
| | Breast carcinoma (1) | 1 : 8 | 1 : 16 | | | | |
| | Breast carcinoma (3) | | 1 : 4 | | | | |
| | Breast carcinoma (4) | | 1 : 4 | | | | |
| | Cervical carcinoma | | 1 : 4 | | | | |
| | Colon carcinoma | 1 : 8 | 1 : 8 | 1 : 16 | | 1 : 4 | |
| | Melanoma | 1 : 16 | 1 : 4 | 1 : 32 | | 1 : 4 | |
| | Ovarian carcinoma (1) | 1 : 8 | | | | | |
| | Ovarian carcinoma (2) | | 1 : 4 | | | | |
| | Fibroadenoma | | —ve | | | —ve | |
| | Pleural cells (1) | —ve | 1 : 2 | 1 : 4 | | —ve | 1 : 8 |
| | Pleural cells (2) | 1 : 2 | 1 : 2 | 1 : 8 | | | 1 : 8 |
| Pleural cells (2) and Pleural cells (1) | Pleural cells (1) | | | —ve | | | —ve |
| | Breast carcinoma (1) | | | 1 : 8 | | | |
| | Melanoma | | | 1 : 4 | | | |
| | Ovarian carcinoma (1) | | | 1 : 8 | | | |
| | Fibroadenoma | | | | | | —ve |
| Ovarian carcinoma | Ovarian carcinoma (1) | | | —ve | | | —ve |
| | Breast carcinoma (1) | | | —ve | | | |
| | Cervical carcinoma | | | | | | —ve |
| | Melanoma | | | —ve | | | |

FIG. 3.—Indirect immunofluorescence. The photograph on the left is phase contrast; the one on the right is of the same cell in UV light. Membrane fluorescence can be seen. Targets are melanoma cells.

cells. In each instance, the binding of the antisera was not specific for the immunizing tumour-cell type, but binding to similar titres with other tumour cells was found (Tables II, III and IV). Although titres were low, membrane immunofluorescence was bright and the entire cell population was stained. The counts obtained in the $^{125}$I-SpA assay were quite high (see Fig. 4).

*Absorption of residual activity for tumour cells by unrelated tumour cells*

The fact that various tumour cells reacted with the same sera suggested that common antigens may be being detected, and this was confirmed by showing that absorption of anti-melanoma serum with colon carcinoma or ovarian carcinoma cells removed all activity to both tumours as well as to other tumour-cell types (Tables II, III and IV). Similarly, absorption of anti-breast-carcinoma sera with ovarian carcinoma cells gave us comparable results. In the case of anti-melanoma serum (Batch 3) where some reactivity to normal cells was still present prior to absorption with unrelated tumour cells, all reactivity to normal and tumour cells was removed.

### DISCUSSION

In this study we have employed primary cell cultures so as to avoid problems that are associated with use of established cell lines, such as contamination with Hela cells (Grimwade, 1976) viral contamination and loss of antigenicity through prolonged culture (Levey, 1973). Our primary cell cultures have limited proliferative potential and so, in order to allow experiments over a

FIG. 4.—$^{125}$I-staphylococcal protein A assay. Melanoma cells were used as targets, anti-melanoma serum at various stages of absorption are shown. The upper curve is the unabsorbed serum diluted 1 : 5 initially to make it directly comparable with the absorbed sera. The middle curve is the antiserum after 35 absorptions on Chang monolayers. The lower curve is anti-melanoma serum absorbed with Chang ×27, foetal fibroblasts ×7, fibroadenoma cells ×2 and colon carcinoma ×3. The control was the normal pre-immunized rabbit serum at a dilution of 1 : 10, which is directly comparable with the lowest dilution of the antisera. Each point on the curve is shown ±s.e. of its arithmetical mean from triplicate samples.

period of time, we have cryopreserved cells, preferably after only one or two passages. This technique has permitted the study of a given primary culture over many months. In these studies, the primary neoplastic cell cultures used for raising antisera were selected for two reasons. Firstly, because of their initial high proliferative capacity, large numbers of cells could be obtained. In some cultures, initial low growth rates were observed and in some cases this could be related to the patient's previous chemotherapy. Secondly, a range of cultures from different primaries was selected, namely colon carcinoma, breast carcinoma, ovarian carcinoma and malignant melanoma, in order that tumour-associated antigens which might be histogenically determined could be studied. A variety of normal cells were chosen as controls for the following reasons:

(i) Foetal fibroblasts were used in an attempt to exclude the presence of common embryonic and tumour antigens. It has been shown that tumour cells have antigens on their cell surfaces which are common to embryonic tissues but are not found on adult cells. Furthermore, these embryonic antigens are able to induce cytotoxic "T" cells and the production of antibody which can block the cytotoxic action of the "T" cells (Hellstrom and Hellstrom, 1975). Cross reactivity between tumours of the same histological type may be due to the expression of embryonic antigens (Baldwin and Embleton, 1974).

(ii) Benign fibroadenoma cells were included as there is evidence that there is antigenic cross reactivity between benign and malignant breast tumours as assessed by the microcytotoxicity assay (Avis, Mosonov and Haughton, 1974).

(iii) Normal fibroblasts were cultured from epimysium obtained from a patient with no evidence of malignancy as a control for "normal" adult human antigens.

(iv) Parietal pleural cells were obtained at *post mortem* from recently deceased men with no evidence of malignant disease. Pleural cells are an important control because it has been claimed that cultures from malignant effusions contain large numbers of mesothelial cells, sometimes to the total exclusion of tumour cells (Cailleau *et al.*, 1974; Whitehead and Hughes, 1975). Unfortunately there is, as yet, no generally applicable technique to ascertain whether a given human cell culture is tumorous or not, and our attempts to grow these "tumour" cells in nude mice have been unsuccessful.

Thus, any conclusions about the nature of cells obtained from malignant effusions is still speculative.

In order to minimize the immunogenicity of "normal" human antigens in the rabbit, target cells were coated with anti-lymphocyte serum (ALS) as originally described by Weiner, Hubbard and Mardiney in 1972. Our results show that the technique does not avoid antibodies to such antigens, since we found high antibody titres against normal cells. Success has been claimed in defining a leukaemia-specific antigen by injecting ALS coated acute lymphoblastic leukaemia cells into rabbits (Brown *et al.*, 1975) but whether this success is due to antibody coating is not known.

We have, therefore, had to use absorption techniques to remove unwanted antibodies. The use of cell monolayers as an immunoabsorbent has several advantages over the more conventional methods using cell pellets. Thus, antisera are exposed only to the surfaces of viable cells and several other potential problems are avoided. The use of fixed cells may lead to alterations of antigenic determinants. If cell suspensions of naturally adherent cells are used, they may not express some antigens that are expressed in the adherent state, and if cell homogenates are used they will contain cytoplasmic products some of which are proteolytic. Finally, in this method, cells are not subjected to trypsinization, and so lengthy incubation periods to allow the regeneration of surface antigens are avoided. The necessity to absorb with foetal calf serum is also obviated, because the monolayers used for absorption are grown in culture medium containing serum some of which will be incorporated into cell membranes (Irie, Irie and Morton, 1976). Sera can undergo multiple absorptions with minimal dilution by this method, which, coupled with the other disadvantages of pelleting techniques, led us to abandon the latter after a preliminary trial.

Both IF and SpA assays have been used in this work to complement each other. IF, though sensitive and allowing the detection of small numbers of positive cells in a population, which is an advantage, particularly if the homogeneity of the population is in question, does however have the disadvantages of subjectivity in interpretation and difficulty in quantitation. The $^{125}$I-SpA assay is objective and semiquantitative and allows detection of IgG subclasses 1, 2 and 4 (Kronvall and Williams, 1969). The lack of detection of other classes has not been a problem, as judged by the concordance of results obtained in the two assay systems, presumably because the majority of antibodies binding to the target cells are IgG.

Chang cells, which are derived from normal liver, are a convenient primary immunoabsorbent, because they are adherent and proliferate rapidly. Thus large numbers can be obtained readily. However, their use could be criticized, because they are an established cell line and so one may expect "tumour-associated" antigens related to continuous proliferative potential. While we cannot exclude this possibility, they are histogenically distinct from our tumour cells used for immunization and so it is unlikely that antibodies to tissue-related tumour antigens would be removed.

Our experiments show that most of the antibody response elicited by tumour cells in rabbits is directed against antigens present on non-tumour cells. Following absorption with Chang cells, the reactivity to the immunizing tumour decreased by about 50-fold. Subsequent absorptions with Chang cells did not further reduce this reactivity, indicating that the remaining activity was against antigens not present on Chang cells. Much of this reactivity was directed against other non-malignant cells such as pleural cells, fibroblasts, benign-breast-tumour cells (all from adult tissues) and foetal fibroblasts. This reactivity was removed by sequential absorptions with these cells. However, residual reactivity remained towards the immunizing tumour cells and to other unrelated tumours. Though the titres were low, definite staining was present with IF, and significant counts were recorded in the $^{125}$I-SpA assay (up to 14× control values). The remaining reactivity to the tumour cells was completely removed by absorption with a tumour other than the immunizing tumour. These results indicate that all tumour cells grown from effusions have a common antigenicity, irrespective of their tissue of origin.

To ensure that the absorption with foetal fibroblasts had not removed antibodies to foetal antigens which may be responsible for the histogenic pattern of human tumour antigens described by other workers, anti-melanoma serum (Batch 3) was absorbed with Chang and pleural cells (1) (see Table III). The serum remained reactive to the tumours and some normal cells at this stage. The serum was then absorbed with colon carcinoma, and this removed all reactivity to tumour and non-tumour cells tested. The experiment indicates firstly, that absorption with foetal fibroblasts is not responsible for the failure to detect histogenically-determined antigens in the previous experiments, and secondly, the great similarity between the set of antigens on the colon carcinoma cells and the set on the melanoma cells.

Other workers have claimed that tumour cells share common tumour antigens (Dickinson, Smith and Dyson, 1976; Seibert *et al.*, 1977; Grimm *et al.*, 1976) and our results are more in keeping with this interpretation rather than the histogenic model of tumour antigens. However, there is evidence, especially in the case of melanoma, that histogenically determined tumour-specific antigens are present (McCoy *et al.*, 1975; Stuhlmiller and Seigler, 1975; Shiku *et al.*, 1976) but differences in approach make direct comparisons difficult.

There are several possible reasons why histogenic tumour-specific antigens were not detected in the present study. Firstly, cells used for immunization may be mesothelial rather than tumour cells. If that were the case, the common antigens detected would be mesothelial. However, absorption with normal pleural cells should have removed antibodies to these antigens, whereas in spite of absorption with these cells, the common "tumour activity" remained. Secondly, as the tumour cells were derived from metastatic deposits, they may be less immunogenic than those found on the parent cell population. Thirdly, rabbits may not possess the antibody diversity to recognize the antigens in question, or to discriminate between them as between human isoantigens (Schulman *et al.*, 1964). Goodwin *et al.* (1972) produced heteroantisera to human melanoma in rabbits and claimed to detect melanoma-specific antibodies. However, the technique used could not discriminate between cytoplasmic or membrane antigens, so comparison between this work and ours is difficult. The antibody titres in Goodwin's studies and that of Greaves *et al.* (1975) were low and comparable with those found in this paper after reactivity to normal cells had been lost. However, these titres could still be used as a diagnostic or prognostic tool (Greaves *et al.*, 1975).

In this study, we have shown that in 2/4 cases, antibodies binding to tumour-cell surfaces can be detected in rabbit antisera after extensive absorption with "normal" non-tumour cells. This variability may reflect differences between the tumour cells used for immunization or may be due to variability in responses on the part of different rabbits. While the exact nature of the common reactivity to tumour cells found in these experiments has yet to be determined, the availability of these antisera permits further investigation of cells in solid tumours and fresh effusions. Furthermore, the methodology described here should be of general application in the analysis of other systems such as the analysis of sera of cancer patients.

The authors would like to thank the following: Mr E. Alderson and Mrs E. Matheson for their technical help, Mr J. B. Layfield for producing the photographs and Mrs S. Pearce for typing the manuscript. Mr Ross and the Medical and Nursing staff of the Oncology centre Newcastle General Hospital, for providing effusion fluid. This work was supported by a grant from the North of England Cancer Research Campaign.

### REFERENCES

AVIS, F., MOSONOV, I. & HAUGHTON, G. (1974) Antigenic Cross Reactivity between Benign and Malignant Neoplasms of the Human Breast. *J. natn. Cancer Inst.*, **52**, 1041.

BALDWIN, R. W. & EMBLETON, M. J. (1974) Neoantigens on Spontaneous and Carcinogen Induced Rat Tumours Defined by Lymphocytoxicity Assays. *Int. J. Cancer*, **13**, 433.

BROWN, G., CAPELLARO, D. & GREAVES, M. F. (1975) Leukaemia-associated Antigens in Man. *J. natn. Cancer Inst.*, **55**, 1281.

CAILLEAU, R., MACKAY, B., YOUNG, R. K. & REEVES, W. J. (1974) Tissue Culture Studies on Pleural Effusions from Breast Cancer Patients. *Cancer Res.*, **34**, 801.

DICKINSON, J. P., SMITH, J. J. & DYSON, J. E. D. (1976) A Cell Surface Antigen Common to Human Tumours: Detection, Localization and Characterisation. *Biochem. Soc. Trans.*, **4**, 125.

GOODWIN, D. P., HORNUNG, M. O., LEONG, S. P. L. & KREMTZ, E. T. (1972) Immune Responses Induced by Human Malignant Melanoma in the Rabbit. *Surgery*, **72**, 737.

GORSKY, Y., VANKY, F. & SULITZEANU, D. (1976) Isolation from Patients with Breast Cancer of Antibodies Specific for Antigens Associated with Breast Cancer and other Malignant Diseases. *Proc. natn. Acad. Sci., U.S.A.*, **73**, 2101.

GREAVES, M. F., BROWN, G., RAPSON, N. T. & LISTER, T. A. (1975) Antisera to Acute Lymphoblastic Leukaemia Cells. *Clin. Immunol. Immunopath.*, **3**, 514.

GRIMM, E. A., SILVER, H. B., ROTH, J. A., CHEE, D. O., GUPTA, R. K. & MORTON, D. L. (1976) Detection of Tumour Associated Antigen in Human Melanoma Cell Line Supernatants. *Int. J. Cancer*, **17**, 559.

GRIMWADE, S. (1976) Hela Takes Over. *Nature, Lond.*, **259**, 172.

HELLSTROM, K. E. & HELLSTROM, I. (1974*a*) Lymphocyte Mediated Cytotoxicity and Serum Activity to Tumor Antigens. *Adv. Immunol.*, **18**, 209.

HELLSTROM, I. & HELLSTROM, K. E. (1974*b*) Cell Mediated Immune Reactions to Tumour Antigens with Particular Emphasis on Immunity to Human Neoplasms. *Cancer, N.Y.*, **34**, 1461.

HELLSTROM, I. & HELLSTROM, K. E. (1975) Cytotoxic Effect of Lymphocytes from Pregnant Mice on Cultivated Tumor Cells. I. Specificity, Nature of Effector Cells and Blocking by Serum. *Int. J. Cancer*, **15**, 1.

HUNTER, W. M. (1973) Radioimmunoassay. In *Handbook of Experimental Immunology*. Vol. 1, Chapter 17. Oxford, Blackwell

IRIE, R. F., IRIE, K. & MORTON, D. L. (1976) A Membrane Antigen Common to Human Cancer and Fetal Brain Tissues. *Cancer Res.*, **36**, 3510.

KRONVALL, G. & WILLIAMS, R. C. (1969) Differences in Anti-protein A Activity among IgG Subgroups. *J. Immunol.*, **103**, 828.

LEVEY, N. L. (1973) Use of an *In vitro* Microcytotoxicity Test to Assess Human Tumor-Specific Cell Mediated Immunity and its Serum-mediated Abrogation. *Natn. Cancer Inst. Monograph*, **37**, 85.

MCCOY, J. L., JEROME, L. F., DEAN, J. H., PERLIN, E., OLDHAM, R. K., CHAR, D. H., COHEN, M. H., FELIX, E. L. & HERBERMAN, R. B. (1975) Inhibition of Leucocyte Migration by Tumor-associated Antigens in Soluble Extracts of Human Malignant Melanoma. *J. natn. Cancer Inst.*, **55**, 19.

MOHANAKUMAR, T., METZGAR, R. S. & MILLER, D. S. (1974) Human Leukaemia Cell Antigens: Serologic Characterization with Xenoantisera. *J. natn. Cancer Inst.*, **52**, 1435.

MURPHY, W. H. & LANDAU, B. J. (1962) Clonal Variation and Interaction of Cells with Viruses. *Natn. Cancer Inst. Monograph*, **7**, 249.

RICHMAN, A. V. (1976) Immunofluorescent Studies of Benign and Malignant Mammary Tissue. *J. natn. Cancer Inst.*, **57**, 263.

SEIBERT, E., SORG, C., HAPPLE, R. & MACHER, E. (1977) Membrane-associated Antigens of Human Malignant Melanoma. III. Specificity of Human Sera Reacting with Cultured Melanoma Cells. *Int. J. Cancer*, **19**, 172.

SHIKU, H., TAKAHASHI, T., OETTGEN, H. F. & OLD, L. J. (1976) Cell-surface Antigens on Human Malignant Melanoma. II. Serological Typing and Immune Adherence Assays and Definition of Two New Surface Antigens. *J. exp. Med.*, **144**, 873.

SCHULMAN, N. R., MARDER, V. J., HILLER, M. C. & COLLIER, E. M. (1964) Platelet Leucocyte Antigens and their Antibodies: Serological, Physiological and Clinical Studies. *Progr. Hematol.*, **4**, 222.

SOUHAMI, R., OWEN, J. J. T. & SEEGER, R. C. (1974) A Method for Growing, Freeze-storing and Isotopically Labelling Human Tumour Cells for *In vitro* Assays of Tumour Immunity. *J. Immunol. Methods*, **5**, 137.

STUHLMILLER, G. M. & SEIGLER, H. F. (1975) Characterization of a Chimpanzee Anti-human Melanoma Antiserum. *Cancer Res.*, **35**, 2132.

WEINER, R. S., HUBBARD, J. D. & MARDINEY, M. R. (1972) Production of Tumor Specific Antibody in Xenogeneic Host: Use of Blocking Antibody. *J. natn. Cancer Inst.*, **49**, 1063.

WHITEHEAD, R. H. & HUGHES, L. E. (1975) Tissue Culture Studies of Malignant Effusions. *Br. J. Cancer*, **32**, 512.